# 2-Bromo­methyl-*N*-isopropyl-7,8-dimeth­oxy-1,2-dihydro-1,3-oxazolo[3,2-*a*]quinoline-4-carboxamide

**DOI:** 10.1107/S1600536808013378

**Published:** 2008-05-10

**Authors:** Svetlana V. Shishkina, Oleg V. Shishkin, Igor V. Ukrainets, Nataliya L. Bereznyakova, Alexandra A. Davidenko

**Affiliations:** a STC "Institute for Single Crystals", National Academy of Sciences of Ukraine, 60 Lenina ave., Kharkiv 61001, Ukraine; b National University of Pharmacy, 4 Blyukhera ave., Kharkiv 61002, Ukraine

## Abstract

In the title compound, C_18_H_21_BrN_2_O_5_, conjugation between the π-donating N—C—O fragment and the π-withdrawing carbonyl group results in considerable redistribution of the electron density within the dihydropyridinol ring. This effect is also promoted by the formation of an intra­molecular N—H⋯O hydrogen bond. The five-membered heterocycle is disordered over two envelope conformations in a 0.35:0.65 ratio.

## Related literature

For related literature, see: Ukrainets *et al.* (2007*a* ▶,*b* ▶); Bürgi & Dunitz (1994 ▶); Hutcheon & James (1977 ▶).
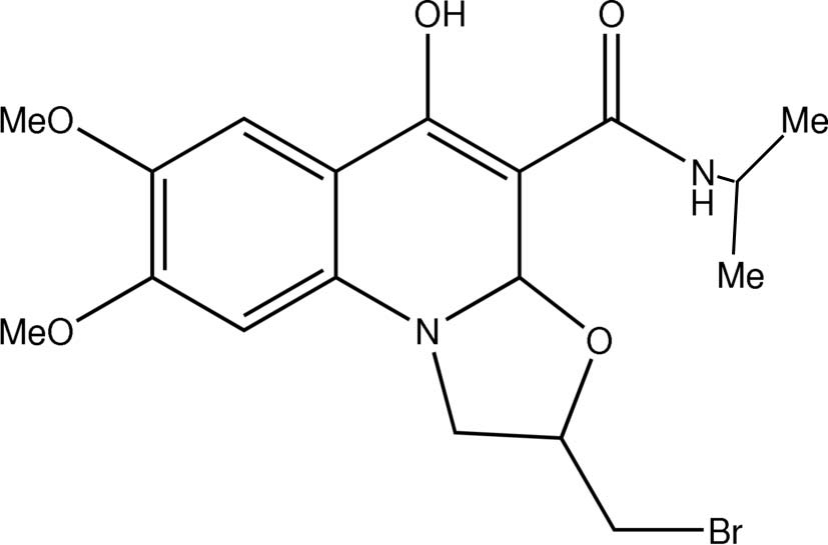


## Experimental

### 

#### Crystal data

C_18_H_21_BrN_2_O_5_
*M* *_r_* = 425.28
Triclinic, 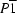

*a* = 8.736 (2) Å
*b* = 9.968 (2) Å
*c* = 10.588 (3) Å
α = 86.90 (2)°
β = 80.90 (2)°
γ = 80.04 (2)°
*V* = 896.4 (4) Å^3^
*Z* = 2
Mo *K*α radiation
μ = 2.33 mm^−1^
*T* = 100 (2) K
0.60 × 0.40 × 0.10 mm

#### Data collection

Oxford Diffraction Xcalibur3 diffractometer
Absorption correction: analytical (Alcock, 1970 ▶) *T*_min_ = 0.287, *T*_max_ = 0.793
6292 measured reflections
3105 independent reflections
2701 reflections with *I* > 2σ(*I*)
*R*_int_ = 0.089

#### Refinement

*R*[*F* ^2^ > 2σ(*F* ^2^)] = 0.066
*wR*(*F* ^2^) = 0.171
*S* = 1.05
3105 reflections
258 parameters
6 restraints
H-atom parameters constrained
Δρ_max_ = 1.76 e Å^−3^
Δρ_min_ = −0.88 e Å^−3^

### 

Data collection: *CrysAlis CCD* (Oxford Diffraction, 2005 ▶); cell refinement: *CrysAlis RED* (Oxford Diffraction, 2005 ▶); data reduction: *CrysAlis RED*; program(s) used to solve structure: *SHELXTL* (Sheldrick, 2008 ▶); program(s) used to refine structure: *SHELXTL*; molecular graphics: *XP* (Siemens, 1998 ▶); software used to prepare material for publication: *SHELXTL*.

## Supplementary Material

Crystal structure: contains datablocks I, global. DOI: 10.1107/S1600536808013378/kp2166sup1.cif

Structure factors: contains datablocks I. DOI: 10.1107/S1600536808013378/kp2166Isup2.hkl

Additional supplementary materials:  crystallographic information; 3D view; checkCIF report

## Figures and Tables

**Table 1:** Hydrogen-bond geometry (Å, °)

*D*—H⋯*A*	*D*—H	H⋯*A*	*D*⋯*A*	*D*—H⋯*A*
N2—H2*A*⋯O3	0.88	1.91	2.634 (5)	138
C10*A*—H10*A*⋯O3^i^	1.00	2.23	3.042	137
C12*A*—H12*A*⋯O2^ii^	0.99	2.33	3.289	164
C12*A*—H12*B*⋯O4^iii^	0.99	2.41	3.173	134
C12*B*—H12*D*⋯O2^ii^	0.99	2.30	3.253	162
C17—H17*A*⋯O5^iv^	0.98	2.41	3.380	172

Symmetry codes: (i) 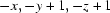; (ii) 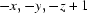; (iii) 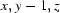; (iv) 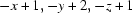.

**Fig. 1.**
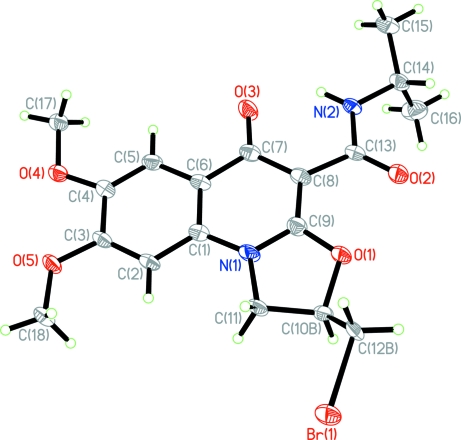
View of the title compound with atomic numbering. All atoms are shown with displacement ellipsoids drawn at the 50% probability level. More predominant orientation (65%) of the disodered fragment of the oxazol ring is shown.

**Fig. 2.**
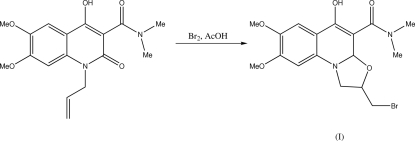
The formation of the title compound.

## supplementary crystallographic information

### Comment

2-Bromomethyl-5-oxo-1,2-dihydro-5*H*-oxazolo[3,2-*a*]quinoline-4- carboxylic acids are labile compounds. Therefore, their amidation are not always successful (Ukrainets *et al.*, 2007*a*; Ukrainets *et al.*, 2007*b*). However the heterocyclization of the previously synthesized *N*—*R*-amides of 1-allyl-4-hydroxy-2-oxo-1,2-dihydroquinoline-3-carboxylic acids was straightforward into the *N*—*R*-amides of oxazolo-quinoline-4-carboxylic acids (I) (Scheme 1). In the present paper, we report the crystal structure of the title compound, (I). The benzpyridone fragment and the O1, C11, O5, O4, O3, C13, O2, N2 atoms are cooplanar whithin 0.02 Å. The C7—O3 (1.263 (5) Å) and C8—C9 (1.386 (6) Å) bonds are elongated comparing to the values in the literature (1.210 Å and 1.326 Å; Burgi & Dunitz, 1994) whereas the C9—O1 (1.336 (5) Å) and N1—C9 (1.333 (6) Å) bonds are shorter than their mean values retrieved from the quoted references (1.354 Å and 1.336 Å). Such redistribution of the electron density can be explained by the conjugation interactions between the N1—C9—O1 π-donating fragment and the C7—O3 π-acceptor carbonyl group. Similar effect was observed earlier in related structure (Hucheon & James, 1977). The formation of the N2—H2N···O3 intramolecular hydrogen bond (Table 1) also promotes the elongation of the carbonyl bond. The five-membered heterocycle ring is disodered over two envelope conformations (A and B) with population A:B 35:65%. The deviation of the C10 atom from the mean plane of the remaining atoms of the ring is -0.41 Å in the conformer A and 0.35 Å in B. The bromomethyl substituent in both conformers is in a pseudo-equatorial orientation (the C9—O1—C10—C12 torsion angle is 145.1 (7) %A in A and -138.1 (5) %A in B). The bromine atom is not disordered and it is located in *ap*-position relatively to the O1—C10 bond in both conformers [the O1—C10—C12—Br1 torsion angle is -179.9 (6) %A (A) and 178.5 (3) %A (B)]. The methoxy groups at the C3 and C4 atoms are almost coplanar to the plane of the aromatic ring (the C18—O5—C3—C2 and C17—O4—C4—C5 torsion angles are 4.2 (6) %A and -6.2 (6) %A, respectively). The isopropyl group has *ap*-conformation relatively to the C8—C13 bond and it is turned away from the C13—N2 bond (the C14—N2—C13—C8 and C13—N2—C14—H14*a* torsion angles are 174.7 (4) %A and -40%A, respectively). In the crystal the molecules of the title compound form the three-dimensional network *via* intermolecular hydrogen bonds (Table 1). The shortened intermolecular contacts H14*a*···Br1^i ^ (i = -*x*,-*y*,1 - *z*) 3.13 Å (van der Waals sum 3.23 Å), H18*c*···C7^ii^ (ii = 1 - *x*,1 - *y*,1 - *z*) 2.70 Å (2.87 Å), Br1···Br1^iii ^(iii = 1 - *x*,-*y*,-*z*) 3.42 Å (3.94 Å) were observed in the crystal. Stacking interaction between parallel aromatic rings is observed [the shortest C3···C1^ii^ (1 - *x*,1 - *y*,1 - *z*) distance is 3.45 Å].

### Experimental

To a stirred solution of the 1-allyl-4-hydroxy-6,7-dimethoxy-2-oxo- 1,2-dihydroquinoline-3-carboxylic acid isopropylamide (3.46 g, 10.0 mmol) in acetic acid (70 ml) was added bromine (0.52 ml, 10.0 mmol) (the solution turned to be colourless). The mixture was diluted with water. The precipitate formed was filtered off, washed with cold water and dried. Yield 3.95 g (93%). m.p. 542–544 K.

### Refinement

All hydrogen atoms were calculated geometrically and included in the refinement in the riding motion approximation with *U*_iso_ constrained to be 1.5 times *U*_eq_ of the carrier atom for the methyl groups and 1.2 times *U*_eq_ of the carrier atom for the other atoms. During refinement the O-C*sp*^3 ^and C*sp*^3-C^*^sp^*3 bonds in the disordered fragment were constrained to 1.44 (1) Å and 1.54 (1) Å, respectively.

### Crystal data

**Table:**

C_18_H_21_BrN_2_O_5_	*Z* = 2
*M**_r_* = 425.28	*F*_000_ = 436
Triclinic, *P*1	*D*_x_ = 1.576 Mg m^−^^3^
*a* = 8.736 (2) Å	Mo *K*α radiation λ = 0.71073 Å
*b* = 9.968 (2) Å	Cell parameters from 2385 reflections
*c* = 10.588 (3) Å	θ = 4–32º
α = 86.90 (2)º	µ = 2.33 mm^−^^1^
β = 80.90 (2)º	*T* = 100 (2) K
γ = 80.04 (2)º	Plate, colourless
*V* = 896.4 (4) Å^3^	0.60 × 0.40 × 0.10 mm

### Data collection

**Table:**

Oxford Diffraction Xcalibur3 diffractometer	3105 independent reflections
Radiation source: Enhance (Mo) X-ray Source	2701 reflections with *I* > 2σ(*I*)
Monochromator: graphite	*R*_int_ = 0.089
Detector resolution: 16.1827 pixels mm^-1^	θ_max_ = 25.0º
*T* = 100(2) K	θ_min_ = 3.3º
ω scans	*h* = −10→10
Absorption correction: analytical(Alcock, 1970)	*k* = −11→11
*T*_min_ = 0.287, *T*_max_ = 0.793	*l* = −12→12
6292 measured reflections	

### Refinement

**Table:**

Refinement on *F*^2^	Secondary atom site location: difference Fourier map
Least-squares matrix: full	Hydrogen site location: inferred from neighbouring sites
*R*[*F*^2^ > 2σ(*F*^2^)] = 0.066	H-atom parameters constrained
*wR*(*F*^2^) = 0.171	*w* = 1/[σ^2^(*F*_o_^2^) + (0.0978*P*)^2^ + 1.3451*P*] where *P* = (*F*_o_^2^ + 2*F*_c_^2^)/3
*S* = 1.05	(Δ/σ)_max_ < 0.001
3105 reflections	Δρ_max_ = 1.76 e Å^−^^3^
258 parameters	Δρ_min_ = −0.88 e Å^−^^3^
6 restraints	Extinction correction: none
Primary atom site location: structure-invariant direct methods	

### Special details

**Table:**

Geometry. All e.s.d.'s (except the e.s.d. in the dihedral angle between two l.s. planes) are estimated using the full covariance matrix. The cell e.s.d.'s are taken into account individually in the estimation of e.s.d.'s in distances, angles and torsion angles; correlations between e.s.d.'s in cell parameters are only used when they are defined by crystal symmetry. An approximate (isotropic) treatment of cell e.s.d.'s is used for estimating e.s.d.'s involving l.s. planes.
Refinement. Refinement of *F*^2^ against ALL reflections. The weighted *R*-factor *wR* and goodness of fit *S* are based on *F*^2^, conventional *R*-factors *R* are based on *F*, with *F* set to zero for negative *F*^2^. The threshold expression of *F*^2^ > σ(*F*^2^) is used only for calculating *R*-factors(gt) *etc*. and is not relevant to the choice of reflections for refinement. *R*-factors based on *F*^2^ are statistically about twice as large as those based on *F*, and *R*- factors based on ALL data will be even larger.

### Fractional atomic coordinates and isotropic or equivalent isotropic displacement parameters (Å^2^)

**Table:**

	*x*	*y*	*z*	*U*_iso_*/*U*_eq_	Occ. (<1)
Br1	0.34636 (6)	0.06689 (5)	0.11180 (4)	0.0383 (2)	
N1	0.2519 (5)	0.3824 (4)	0.4324 (3)	0.0265 (8)	
N2	−0.0961 (4)	0.3493 (4)	0.8290 (3)	0.0225 (8)	
H2A	−0.0854	0.4337	0.8407	0.027*	
O1	0.1378 (4)	0.2004 (3)	0.4688 (3)	0.0292 (7)	
O2	−0.0441 (4)	0.1744 (3)	0.6927 (3)	0.0308 (8)	
O3	0.0389 (4)	0.5646 (3)	0.7631 (3)	0.0298 (7)	
O4	0.3952 (4)	0.8815 (3)	0.5332 (3)	0.0256 (7)	
O5	0.5279 (4)	0.7664 (3)	0.3238 (3)	0.0262 (7)	
C1	0.2878 (5)	0.5081 (4)	0.4564 (4)	0.0222 (9)	
C2	0.3940 (5)	0.5707 (4)	0.3692 (4)	0.0228 (9)	
H2B	0.4432	0.5282	0.2916	0.027*	
C3	0.4256 (5)	0.6961 (4)	0.3988 (4)	0.0216 (9)	
C4	0.3527 (5)	0.7586 (4)	0.5161 (4)	0.0233 (9)	
C5	0.2488 (5)	0.6966 (4)	0.5983 (4)	0.0243 (9)	
H5A	0.1987	0.7400	0.6753	0.029*	
C6	0.2142 (5)	0.5693 (4)	0.5715 (4)	0.0226 (9)	
C7	0.1030 (5)	0.5046 (5)	0.6613 (4)	0.0237 (9)	
C8	0.0756 (5)	0.3709 (4)	0.6301 (4)	0.0223 (9)	
C9	0.1530 (5)	0.3193 (4)	0.5138 (4)	0.0239 (9)	
C10A	0.1932 (13)	0.2038 (11)	0.3329 (6)	0.034 (4)	0.352 (14)
H10A	0.1049	0.2361	0.2832	0.041*	0.352 (14)
C12A	0.2721 (17)	0.0571 (12)	0.2995 (14)	0.034 (4)	0.352 (14)
H12A	0.1958	−0.0067	0.3198	0.040*	0.352 (14)
H12B	0.3614	0.0269	0.3471	0.040*	0.352 (14)
C10B	0.2633 (7)	0.1653 (5)	0.3608 (5)	0.0207 (18)	0.648 (14)
H10B	0.3527	0.0973	0.3854	0.025*	0.648 (14)
C12B	0.1808 (8)	0.1107 (7)	0.2615 (5)	0.0202 (19)	0.648 (14)
H12C	0.0939	0.1804	0.2379	0.024*	0.648 (14)
H12D	0.1376	0.0282	0.2954	0.024*	0.648 (14)
C11	0.3126 (5)	0.3038 (4)	0.3165 (4)	0.0264 (10)	
H11B	0.4217	0.2559	0.3170	0.032*	0.352 (14)
H11A	0.3070	0.3614	0.2376	0.032*	0.352 (14)
H11C	0.4281	0.2960	0.2944	0.032*	0.648 (14)
H11D	0.2623	0.3439	0.2425	0.032*	0.648 (14)
C13	−0.0266 (5)	0.2875 (4)	0.7177 (4)	0.0219 (9)	
C14	−0.1879 (5)	0.2772 (5)	0.9286 (4)	0.0259 (9)	
H14A	−0.2515	0.2222	0.8880	0.031*	
C15	−0.2986 (6)	0.3822 (5)	1.0142 (5)	0.0344 (11)	
H15A	−0.3679	0.4416	0.9627	0.052*	
H15B	−0.2369	0.4371	1.0534	0.052*	
H15C	−0.3621	0.3353	1.0813	0.052*	
C16	−0.0800 (7)	0.1829 (6)	1.0052 (5)	0.0425 (13)	
H16A	−0.0101	0.1164	0.9485	0.064*	
H16B	−0.1428	0.1350	1.0720	0.064*	
H16C	−0.0169	0.2362	1.0449	0.064*	
C17	0.3101 (5)	0.9556 (4)	0.6430 (4)	0.0260 (9)	
H17A	0.3497	1.0412	0.6470	0.039*	
H17B	0.3248	0.9005	0.7210	0.039*	
H17C	0.1980	0.9754	0.6356	0.039*	
C18	0.6008 (5)	0.7119 (5)	0.2022 (4)	0.0263 (9)	
H18A	0.6710	0.7723	0.1586	0.039*	
H18B	0.5196	0.7050	0.1499	0.039*	
H18C	0.6615	0.6213	0.2151	0.039*	

### Atomic displacement parameters (Å^2^)

**Table:**

	*U*^11^	*U*^22^	*U*^33^	*U*^12^	*U*^13^	*U*^23^
Br1	0.0475 (4)	0.0419 (4)	0.0227 (3)	−0.0146 (2)	0.0145 (2)	−0.0105 (2)
N1	0.030 (2)	0.033 (2)	0.0146 (17)	−0.0092 (15)	0.0090 (15)	−0.0082 (15)
N2	0.0215 (18)	0.0273 (19)	0.0159 (17)	−0.0045 (14)	0.0062 (14)	−0.0004 (14)
O1	0.0334 (18)	0.0305 (17)	0.0208 (16)	−0.0113 (13)	0.0137 (13)	−0.0070 (13)
O2	0.0341 (18)	0.0302 (17)	0.0258 (16)	−0.0107 (13)	0.0104 (14)	−0.0068 (13)
O3	0.0322 (17)	0.0343 (17)	0.0224 (16)	−0.0134 (13)	0.0074 (14)	−0.0053 (13)
O4	0.0254 (16)	0.0266 (15)	0.0233 (16)	−0.0063 (12)	0.0047 (13)	−0.0056 (13)
O5	0.0280 (16)	0.0304 (16)	0.0191 (15)	−0.0113 (13)	0.0077 (13)	−0.0023 (12)
C1	0.020 (2)	0.027 (2)	0.019 (2)	−0.0027 (16)	−0.0005 (17)	−0.0025 (17)
C2	0.020 (2)	0.031 (2)	0.0145 (19)	−0.0028 (16)	0.0039 (16)	−0.0006 (17)
C3	0.018 (2)	0.029 (2)	0.016 (2)	−0.0027 (16)	0.0026 (16)	0.0019 (17)
C4	0.020 (2)	0.028 (2)	0.021 (2)	−0.0046 (16)	−0.0007 (17)	−0.0006 (17)
C5	0.023 (2)	0.027 (2)	0.021 (2)	−0.0021 (16)	0.0023 (17)	−0.0060 (17)
C6	0.018 (2)	0.030 (2)	0.018 (2)	−0.0022 (16)	0.0017 (17)	−0.0033 (17)
C7	0.021 (2)	0.031 (2)	0.018 (2)	−0.0030 (16)	−0.0004 (17)	−0.0033 (17)
C8	0.023 (2)	0.031 (2)	0.0118 (19)	−0.0037 (16)	0.0018 (17)	−0.0010 (16)
C9	0.023 (2)	0.028 (2)	0.021 (2)	−0.0063 (17)	−0.0005 (18)	−0.0028 (17)
C10A	0.029 (9)	0.038 (9)	0.034 (9)	−0.010 (7)	0.002 (7)	0.003 (7)
C12A	0.030 (8)	0.040 (9)	0.031 (8)	−0.011 (7)	0.002 (6)	−0.017 (6)
C10B	0.020 (4)	0.020 (4)	0.018 (4)	0.002 (3)	0.003 (3)	0.003 (3)
C12B	0.016 (4)	0.031 (4)	0.016 (3)	−0.013 (3)	0.002 (3)	−0.004 (3)
C11	0.028 (2)	0.032 (2)	0.018 (2)	−0.0109 (18)	0.0104 (18)	−0.0082 (18)
C13	0.017 (2)	0.027 (2)	0.019 (2)	−0.0014 (16)	0.0020 (17)	−0.0020 (17)
C14	0.025 (2)	0.032 (2)	0.018 (2)	−0.0069 (18)	0.0061 (18)	−0.0017 (17)
C15	0.028 (2)	0.040 (3)	0.030 (3)	−0.002 (2)	0.009 (2)	−0.005 (2)
C16	0.048 (3)	0.038 (3)	0.031 (3)	0.006 (2)	0.008 (2)	0.006 (2)
C17	0.025 (2)	0.027 (2)	0.024 (2)	−0.0041 (17)	0.0018 (18)	−0.0041 (18)
C18	0.023 (2)	0.035 (2)	0.019 (2)	−0.0080 (18)	0.0050 (18)	0.0002 (18)

### Geometric parameters (Å, °)

**Table:**

Br1—C12B	1.981 (6)	C10A—C12A	1.539 (5)
Br1—C12A	1.99 (1)	C10A—C11	1.547 (5)
N1—C9	1.334 (6)	C10A—H10A	1.000
N1—C1	1.388 (6)	C12A—H12A	0.990
N1—C11	1.469 (5)	C12A—H12B	0.990
N2—C13	1.363 (5)	C10B—C12B	1.535 (5)
N2—C14	1.457 (5)	C10B—C11	1.544 (4)
N2—H2A	0.880	C10B—H10B	1.000
O1—C9	1.336 (5)	C12B—H12C	0.990
O1—C10A	1.445 (5)	C12B—H12D	0.990
O1—C10B	1.465 (4)	C11—H11B	0.990
O2—C13	1.212 (5)	C11—H11A	0.990
O3—C7	1.263 (5)	C11—H11C	0.990
O4—C4	1.369 (5)	C11—H11D	0.990
O4—C17	1.442 (5)	C14—C16	1.511 (7)
O5—C3	1.361 (5)	C14—C15	1.525 (6)
O5—C18	1.433 (5)	C14—H14A	1.000
C1—C6	1.403 (6)	C15—H15A	0.980
C1—C2	1.403 (6)	C15—H15B	0.980
C2—C3	1.387 (6)	C15—H15C	0.980
C2—H2B	0.950	C16—H16A	0.980
C3—C4	1.426 (6)	C16—H16B	0.980
C4—C5	1.360 (6)	C16—H16C	0.980
C5—C6	1.408 (6)	C17—H17A	0.980
C5—H5A	0.950	C17—H17B	0.980
C6—C7	1.456 (6)	C17—H17C	0.980
C7—C8	1.458 (6)	C18—H18A	0.980
C8—C9	1.386 (6)	C18—H18B	0.980
C8—C13	1.503 (6)	C18—H18C	0.980
			
C9—N1—C1	122.8 (4)	C10B—C12B—H12C	110.7
C9—N1—C11	111.5 (3)	Br1—C12B—H12C	110.7
C1—N1—C11	125.6 (3)	C10B—C12B—H12D	110.7
C13—N2—C14	120.8 (4)	Br1—C12B—H12D	110.7
C13—N2—H2A	119.6	H12C—C12B—H12D	108.8
C14—N2—H2A	119.6	N1—C11—C10B	100.3 (3)
C9—O1—C10A	107.1 (4)	N1—C11—C10A	98.6 (4)
C9—O1—C10B	108.5 (3)	N1—C11—H11B	112.0
C4—O4—C17	115.8 (3)	C10B—C11—H11B	85.6
C3—O5—C18	118.0 (3)	C10A—C11—H11B	112.0
N1—C1—C6	117.2 (4)	N1—C11—H11A	112.0
N1—C1—C2	121.3 (4)	C10B—C11—H11A	134.1
C6—C1—C2	121.5 (4)	C10A—C11—H11A	112.0
C3—C2—C1	118.6 (4)	H11B—C11—H11A	109.7
C3—C2—H2B	120.7	N1—C11—H11C	111.7
C1—C2—H2B	120.7	C10B—C11—H11C	111.7
O5—C3—C2	124.4 (4)	C10A—C11—H11C	135.7
O5—C3—C4	115.1 (4)	H11A—C11—H11C	86.1
C2—C3—C4	120.4 (4)	N1—C11—H11D	111.7
C5—C4—O4	125.9 (4)	C10B—C11—H11D	111.7
C5—C4—C3	119.9 (4)	C10A—C11—H11D	86.6
O4—C4—C3	114.2 (4)	H11B—C11—H11D	128.5
C4—C5—C6	121.2 (4)	H11C—C11—H11D	109.5
C4—C5—H5A	119.4	O2—C13—N2	122.8 (4)
C6—C5—H5A	119.4	O2—C13—C8	123.1 (4)
C1—C6—C5	118.4 (4)	N2—C13—C8	114.2 (4)
C1—C6—C7	121.4 (4)	N2—C14—C16	110.1 (4)
C5—C6—C7	120.2 (4)	N2—C14—C15	108.4 (4)
O3—C7—C6	118.9 (4)	C16—C14—C15	110.9 (4)
O3—C7—C8	123.4 (4)	N2—C14—H14A	109.1
C6—C7—C8	117.7 (4)	C16—C14—H14A	109.1
C9—C8—C7	116.5 (4)	C15—C14—H14A	109.1
C9—C8—C13	119.8 (4)	C14—C15—H15A	109.5
C7—C8—C13	123.7 (4)	C14—C15—H15B	109.5
N1—C9—O1	111.4 (4)	H15A—C15—H15B	109.5
N1—C9—C8	124.2 (4)	C14—C15—H15C	109.5
O1—C9—C8	124.4 (4)	H15A—C15—H15C	109.5
O1—C10A—C12A	105.8 (8)	H15B—C15—H15C	109.5
O1—C10A—C11	104.2 (4)	C14—C16—H16A	109.5
C12A—C10A—C11	112.4 (9)	C14—C16—H16B	109.5
O1—C10A—H10A	111.4	H16A—C16—H16B	109.5
C12A—C10A—H10A	111.4	C14—C16—H16C	109.5
C11—C10A—H10A	111.4	H16A—C16—H16C	109.5
C10A—C12A—Br1	104.3 (8)	H16B—C16—H16C	109.5
C10A—C12A—H12A	110.9	O4—C17—H17A	109.5
Br1—C12A—H12A	110.9	O4—C17—H17B	109.5
C10A—C12A—H12B	110.9	H17A—C17—H17B	109.5
Br1—C12A—H12B	110.9	O4—C17—H17C	109.5
H12A—C12A—H12B	108.9	H17A—C17—H17C	109.5
O1—C10B—C12B	104.0 (4)	H17B—C17—H17C	109.5
O1—C10B—C11	103.4 (3)	O5—C18—H18A	109.5
C12B—C10B—C11	111.4 (5)	O5—C18—H18B	109.5
O1—C10B—H10B	112.4	H18A—C18—H18B	109.5
C12B—C10B—H10B	112.4	O5—C18—H18C	109.5
C11—C10B—H10B	112.4	H18A—C18—H18C	109.5
C10B—C12B—Br1	105.1 (4)	H18B—C18—H18C	109.5
			
C9—N1—C1—C6	−0.5 (7)	C1—N1—C9—C8	0.0 (7)
C11—N1—C1—C6	178.1 (4)	C11—N1—C9—C8	−178.8 (4)
C9—N1—C1—C2	179.2 (4)	C10A—O1—C9—N1	−18.1 (7)
C11—N1—C1—C2	−2.2 (7)	C10B—O1—C9—N1	13.6 (5)
N1—C1—C2—C3	−179.7 (4)	C10A—O1—C9—C8	161.7 (7)
C6—C1—C2—C3	−0.1 (7)	C10B—O1—C9—C8	−166.6 (5)
C18—O5—C3—C2	4.1 (6)	C7—C8—C9—N1	1.7 (7)
C18—O5—C3—C4	−177.6 (4)	C13—C8—C9—N1	−176.6 (4)
C1—C2—C3—O5	178.9 (4)	C7—C8—C9—O1	−178.1 (4)
C1—C2—C3—C4	0.7 (6)	C13—C8—C9—O1	3.6 (7)
C17—O4—C4—C5	−6.1 (6)	C9—O1—C10A—C12A	145.3 (7)
C17—O4—C4—C3	172.8 (4)	C9—O1—C10A—C11	26.6 (9)
O5—C3—C4—C5	−179.8 (4)	O1—C10A—C12A—Br1	−180.0 (6)
C2—C3—C4—C5	−1.4 (7)	C11—C10A—C12A—Br1	−66.9 (9)
O5—C3—C4—O4	1.2 (6)	C9—O1—C10B—C12B	−138.0 (5)
C2—C3—C4—O4	179.6 (4)	C9—O1—C10B—C11	−21.5 (5)
O4—C4—C5—C6	−179.6 (4)	O1—C10B—C12B—Br1	178.5 (3)
C3—C4—C5—C6	1.6 (7)	C11—C10B—C12B—Br1	67.7 (5)
N1—C1—C6—C5	179.8 (4)	C9—N1—C11—C10B	−13.9 (5)
C2—C1—C6—C5	0.2 (7)	C1—N1—C11—C10B	167.4 (5)
N1—C1—C6—C7	−0.9 (6)	C9—N1—C11—C10A	14.8 (7)
C2—C1—C6—C7	179.5 (4)	C1—N1—C11—C10A	−164.0 (6)
C4—C5—C6—C1	−0.9 (7)	O1—C10B—C11—N1	20.3 (5)
C4—C5—C6—C7	179.8 (4)	C12B—C10B—C11—N1	131.5 (5)
C1—C6—C7—O3	−179.4 (4)	O1—C10A—C11—N1	−24.0 (8)
C5—C6—C7—O3	−0.1 (7)	C12A—C10A—C11—N1	−138.1 (8)
C1—C6—C7—C8	2.6 (6)	C14—N2—C13—O2	4.3 (7)
C5—C6—C7—C8	−178.2 (4)	C14—N2—C13—C8	−174.8 (4)
O3—C7—C8—C9	179.1 (4)	C9—C8—C13—O2	0.9 (7)
C6—C7—C8—C9	−2.9 (6)	C7—C8—C13—O2	−177.3 (4)
O3—C7—C8—C13	−2.6 (7)	C9—C8—C13—N2	180.0 (4)
C6—C7—C8—C13	175.4 (4)	C7—C8—C13—N2	1.7 (6)
C1—N1—C9—O1	179.8 (4)	C13—N2—C14—C16	79.8 (5)
C11—N1—C9—O1	1.0 (5)	C13—N2—C14—C15	−158.7 (4)

### Hydrogen-bond geometry (Å, °)

**Table:**

*D*—H···*A*	*D*—H	H···*A*	*D*···*A*	*D*—H···*A*
N2—H2A···O3	0.88	1.91	2.634 (5)	138
C10A—H10A···O3^i^	1.00	2.23	3.042	137
C12A—H12A···O2^ii^	0.99	2.33	3.289	164
C12A—H12B···O4^iii^	0.99	2.41	3.173	134
C12B—H12D···O2^ii^	0.99	2.30	3.253	162
C17—H17A···O5^iv^	0.98	2.41	3.380	172

Symmetry codes: (i) −*x*, −*y*+1, −*z*+1; (ii) −*x*, −*y*, −*z*+1; (iii) *x*, *y*−1, *z*; (iv) −*x*+1, −*y*+2, −*z*+1.
